# Measuring Problematic Mobile Phone Use: Development and Preliminary Psychometric Properties of the PUMP Scale

**DOI:** 10.1155/2013/912807

**Published:** 2013-09-04

**Authors:** Lisa J. Merlo, Amanda M. Stone, Alex Bibbey

**Affiliations:** ^1^Department of Psychiatry, University of Florida, P.O. Box 100183, Gainesville, FL 32610-0183, USA; ^2^University of Florida, Gainesville, FL 32610, USA; ^3^Department of Emergency Medicine, Orlando Regional Medical Center, Orlando, FL 32806, USA; ^4^Department of Radiology, Duke University, Durham, NC 27705, USA

## Abstract

This study aimed to develop and assess the psychometric properties of an English language measure of problematic mobile phone use. Participants were recruited from a university campus, health science center, and other public locations. The sample included 244 individuals (68.4% female) aged 18–75. Results supported a unidimensional factor structure for the 20-item self-report Problematic Use of Mobile Phones (PUMP) Scale. Internal consistency was excellent (*α* = 0.94). Strong correlations (*r* = .76, *P* < .001) were found between the PUMP Scale and an existing scale of cellular phone dependency that was validated in Asia, as well as items assessing frequency and intensity of mobile phone use. Results provide preliminary support for the use of the PUMP Scale to measure problematic use of mobile phones.

## 1. Introduction

Mobile phones (a.k.a., cellular telephones) have many perceived benefits, including increased accessibility and social connection, efficiency in the workplace, convenience, and improved safety. However, in recent years, there has been increasing public interest in the negative consequences of mobile phone use. In one Saudi Arabian study, 44.4% of participants related common health complaints such as headache, trouble concentrating, memory loss, hearing loss, and fatigue to their mobile phone use [1]. Another Saudi Arabian study suggested that 3%-4% of mobile phone users exhibit problems such as tension, fatigue, sleep disturbance, and dizziness related to their mobile phone use, and over 20% complain of headaches [2]. Accidents caused by distracted driving [3, 4] have been highlighted as a public health concern. In addition, anecdotal observation and media reports suggest that the number of self-professed “cell phone addicts” and compulsive users of “crack-berries” and other smartphones has increased as mobile phones have become ubiquitous. Public recognition of this phenomenon is reflected in the many websites and blogs addressing the issue, as well as numerous articles in the popular press describing cell phone addiction. Though stories have appeared in publications such as the *New York Times *[5], the *Los Angeles Times *[6], and http://www.forbes.com/ [7] for many years, the academic literature surrounding problematic mobile phone use remains fairly limited, even when compared to other “behavioral addictions” such as pathological gambling, problematic internet use, and problem video gaming [8–10]. 

While “addiction” is a term commonly used and arguably overused in society, the conceptualization of addiction remains controversial even among researchers and clinicians who specialize in substance use disorders and addictive behaviors. Indeed, the *Diagnostic and Statistical Manual of Mental Disorders, Fourth Edition—Text Revision *[*DSM-IV-TR*] [11] did not include a condition called “addiction.” Rather, it described substance abuse and substance dependence as distinct psychiatric disorders, and failed to include discussion of addictive behaviors that do not involve substance use. Furthermore, the recently released *Diagnostic and Statistical Manual of Mental Disorders, Fifth Edition* (*DSM-5*) describes “substance use disorders” using the following 11 criteria: (1) use in larger quantities or over longer amounts of time than initially intended, (2) a desire to cut down or control use, (3) spending a great deal of time obtaining, using, or recovering from the substance, (4) craving, (5) recurrent substance use resulting in a failure to fulfill major role obligations, (6) continued use despite social/interpersonal problems, (7) neglect of other important activities because of substance use, (8) use in situations in which it is physically hazardous, (9) continued use of the substance despite adverse physical or psychological consequences associated with use, (10) tolerance, and (11) withdrawal symptoms [12]. 

Though the *DSM-IV-TR* and *DSM-5* do not include any disorders related to the problematic use of technology, pathological gambling is included in *DSM-IV* as a diagnosable condition under the category of impulse control disorders not elsewhere classified [11], and in *DSM-5 *as the first “behavioral addiction.” Even though pathological gambling does not involve the use of a chemical substance, the similarities between the diagnostic criteria for substance use disorders and pathological gambling are striking. In general terms, both may be described as disorders involving loss of control over a compulsive, time- and resource-consuming behavior, which persists in the face of adverse consequences, with continued escalation of the behavior and/or withdrawal symptoms from reduction of the behavior. 

Similarly, it was suggested as early as 1982 (i.e., well before the widespread use of mobile phones) that pathological use of technology may exist in the form of “technodependence” [13]. The constructs of internet addiction and problem video gaming are gaining both clinical and empirical support [10, 14]. In addition, though problematic mobile phone use has not, to date, been recognized as a diagnosable condition, experts in the field are debating its inclusion as one [15]. While evidence is scarce regarding a true “addiction” to mobile phones, data from recent studies suggest that some mobile phone users exhibit serious problematic behaviors analogous to the diagnostic criteria for substance use disorders or pathological gambling. These symptoms include preoccupation with mobile phone-based communication, excessive time or money spent on mobile telephones/communication plans, use of cellular devices in socially inappropriate or even physically dangerous situations (e.g., “texting” while driving an automobile), adverse effects on relationships, increased frequency or duration of mobile phone communication, and anxiety when separated from one's telephone or when without an adequate cellular signal [16–19]. Given these findings, it seems plausible that the consequences and psychological dependence seen in problematic mobile phone use (like pathological gambling and problematic internet and video game use) seem to parallel substance use and dependence and may be important to consider as a potential diagnostic entity and target of intervention. 

In order to evaluate the extent to which problematic mobile phone use may be related to other addictive behaviors, research is needed to clarify the construct. To date, research on problematic mobile phone use has been limited by the lack of validated diagnostic criteria or standardized assessment measures. For this study, we operationally defined “problematic mobile phone use” as any pattern of mobile phone use resulting in subjective distress or impairment in important areas of functioning. Given that some individuals have legitimate reason to use their mobile phone very frequently (e.g., for work obligations) and are able to do so without negative consequences, we believed it was important to distinguish “problematic” use from “very frequent” use. We expected rates of mobile phone use to be higher among individuals who exhibited symptoms of problematic mobile phone use, just as substance abusers generally tend to use substances in greater quantities/frequencies than nonabusers. However, as with substance use disorders, we did not feel that high frequency use should be considered a symptom of the condition. For this study, quantity of use was not included as a component of “problematic mobile phone use,” except that individuals' subjective assessment of their use as excessive and troublesome was considered. 

The purpose of the present study was to develop an English language measure of problematic mobile phone use symptoms, based on adaptations of the *DSM-5* substance use disorder criteria. The study followed a similar methodology to that utilized in previous studies regarding behavioral addictions [10, 20]. Specifically, our overarching hypotheses for this study included the following.

1. Symptoms of problematic mobile phone use can be measured reliably and validly using a self-report questionnaire.
2. Scores on the preliminary measure of problematic mobile phone use developed for this study will correlate significantly with an existing measure of cellular phone “dependency,” which was validated on Asian samples.
3. Symptoms of problematic mobile phone use will correlate positively with frequency and intensity of mobile telephone usage.

## 2. Method

 All procedures were approved by the University of Florida Institutional Review Board. Participants were recruited for this study using several methods. First, flyers advertising the study were posted around the university campus, the health science center, and in public locations including a mobile phone store. Individuals who were interested in study participation called the research team to obtain a survey packet or to arrange a time to complete the survey. Second, university students were recruited for the study via announcements made in various undergraduate and graduate level courses. Questionnaires were passed out with a self-addressed, stamped envelope for participants to return the survey to the research team. Third, other participants learned of the study via word-of-mouth and made contact with members of the research team in order to participate. All surveys were completed anonymously. Completion of the full study questionnaire required 20–30 minutes, and no compensation was provided to study participants.

### 2.1. Participants

Data were collected from 244 individuals (68.4% female) who ranged in age from 18 to 75 years old (*M* = 29.8 years old, SD = 14.1 years). Participants were self-identified as Caucasian (74.4%), Hispanic/Latino (11.3%), Asian/Pacific Islander (9.2%), African American/Black (2.5%), or Other (2.5%). The sample included university students (37.7%), individuals employed full-time (32.6%), individuals employed part-time (17.4%), and individuals who were not currently employed or in school (12.2%). Annual income was reported by 86.5% of the sample and ranged from $0 to $190,000 (*M* = $24,562, SD = $35,587).

### 2.2. Measures

#### 2.2.1. Problematic Use of Mobile Phones (PUMP) Scale

A pool of 69 potential items for the PUMP Scale was developed by the first author based upon (1) informal interviews with several self-identified “cell phone addicts” who contacted the first author to discuss their mobile phone usage, (2) adaptation of the *DSM-IV* criteria for substance use disorders, and (3) review of existing measures assessing consequences of excessive internet use. These items were reviewed by 4 undergraduate research assistants for clarity but were not specifically pretested to assess psychometric properties before the questionnaires were distributed. After reviewing the proposed criteria for substance use disorders in the *DSM-5 *[21], which was not yet published, the first and second authors together selected 22 items for inclusion in the scale. Item selection was guided by the rational method, with the authors together choosing the 2 items that best reflected each of the 11 substance use disorder criteria proposed by the *DSM-5* Task Force on Substance-Related Disorders. All items were rated on a 5-point scale ranging from 1 = “strongly disagree” to 5 = “strongly agree.” Scale analysis was utilized to assess psychometric properties of the individual items and the scale as a whole. Further description of the scale is included in the results section.

#### 2.2.2. Cellular Phone Dependence Tendency Questionnaire (CPDQ: [22])

The CPDQ was originally developed in Japan, to assess cellular phone “dependency” among Japanese university students. It was later translated from Japanese to Thai and was used to study high school and university students in Thailand [18]. Kawasaki and colleagues also published an English translation of the CPDQ in the report of their research [18]. For the present study, some items from this English translation were reworded slightly to more closely match the accepted local vernacular. The CPDQ is a 20-item self-report measure. Though it taps the domain of “cell phone addiction,” the CPDQ was not developed on the basis of either *DSM-IV* or *DSM-5* criteria for substance use disorders and does not include items that would be considered reflective of “abuse” criteria per *DSM-IV-TR* guidelines. Rather, items solely assess respondents' perceived dependence on their cellular phone (e.g., “I would feel worse if I lost my cellular phone than if I lost my wallet” and “I send text messages even during work or class”). Items are rated on a 4-point scale ranging from “not true at all” to “true.” The CPDQ has demonstrated good reliability and validity in non-English-speaking populations [18, 19, 22]. In addition, internal consistency for the current sample was excellent (*α* = .91).

#### 2.2.3. Cellular Phone Usage Questionnaire (CUQ)

The CUQ is a compilation of items assessing specific mobile phone usage patterns. It was developed for the present study as a general measurement of mobile phone use and does not attempt to distinguish excessive usage or identify consequences or symptoms associated with mobile phone use. Items assess the amount of time spent utilizing various components of cellular phones (e.g., phone minutes, text messaging, emailing, internet access, and video game play). Items are rated on a 6-point scale ranging from “never” to “constantly.” Participants also completed 3 self-assessment questions regarding perceptions of their mobile phone usage. Items are included in Table 1.

## 3. Results

 All statistical analyses were conducted using PASW 17.0. In order to address isolated cases of missing data, mean substitution was utilized for subscales in which at least 80% of the data were complete. 

### 3.1. Mobile Phone Use Patterns

 Participants reported having a mobile phone for an average of 7.29 years (SD = 3.73, range = 0–20 years). The majority of respondents (88.8%) reported having a personal cellular phone with a monthly contract, 6.6% reported having a personal cellular phone with a prepaid contract, and 1.2% reported sharing a cellular phone with at least one other person. Of the sample, 3.3% of respondents denied having a cellular phone. 

### 3.2. Reliability Analysis

The items of the proposed PUMP Scale were subjected to scale analysis. Results demonstrated that the 2 items assessing the DSM criterion “desire to cut down” (i.e., “I would feel relieved if I was somewhere that my cell phone did not work” and “I sometimes wish I could get rid of my cell phone”) had extremely low item total correlations (.05 and .15, resp.). After first considering their theoretical importance, it was decided that these items should be deleted from the final scale, as they did not appear to fit the overall construct of problematic mobile phone use. The final PUMP Scale demonstrated excellent internal consistency (20 items, *α* = .94). Removal of any item would have resulted in a negative impact on the scale alpha. Items included in the final PUMP Scale are listed in Table 2. 

### 3.3. Factorial Validity

 A principal components analysis was utilized to assess the factor structure of the 20-item PUMP Scale. Results supported a one-factor solution, with factor loadings for all items ≥.48. The one-factor solution explained 49.05% of the variance, meeting Carmines and Zeller's criterion [23]. Analysis of the Scree plot also supported a one-factor solution [24], with the eigenvalue of the first component (9.86) far exceeding the eigenvalue of the second component (1.45), which was not significantly different from the remaining eigenvalues.

### 3.4. Convergent and Discriminant Validity Data

Scores on the PUMP Scale ranged from 20 to 82 (*M* = 38.40, SD = 16.11) out of a possible score of 100. Frequency counts for each item response are listed in Table 3. PUMP scores were compared to scores on the Cellular Phone Dependency Tendency Questionnaire (CPDQ), the Cell Phone Use Questionnaire (CUQ), and the self-assessment items. Results are listed in Table 1. It is noteworthy that PUMP Scale total scores were not associated with the length of time the individual has owned a mobile phone (Pearson *r* = −.08, ns) or with the amount of money spent per month for mobile phone minutes (Pearson *r* = −.04, ns). However, PUMP Scale scores were positively correlated with the amount of time spent engaging in any form of mobile phone use (see Table 1), as well as the amount of money spent for text messaging service (*r* = .27, *P* < .001). As seen in Table 1, PUMP Scale scores also correlated positively with perceptions of excessive mobile phone usage, including self-reported feelings of “addiction” to the mobile phone.

## 4. Discussion

The purpose of the present study was to develop and validate a self-report measure of problematic mobile phone use (i.e., “cell phone addiction”) for English-speaking respondents, based on criteria utilized for other addictive behaviors. Results indicated that problematic mobile phone use can be measured via self-report. The Problematic Use of Mobile Phones (PUMP) Scale demonstrated a single-factor structure, with excellent internal consistency. It also displayed convergent validity when compared to an existing measure of cellular phone dependency [the CPDQ [22]], items measuring the frequency and intensity of cellular phone use behaviors (the CUQ), and self-reported feelings of “addiction” to the mobile phone. These data provide preliminary support for the use of the PUMP Scale in research examining problematic mobile phone use in English-speaking samples.

Items included on the PUMP Scale instrument covered a wide range of symptoms. Most participants did not report symptoms, but it is noteworthy that a significant minority of respondents endorsed experiencing harm to their relationships, finances, and safety as a result of excessive phone use or use in inappropriate circumstances. Some respondents also acknowledged subjective loss of control over escalating phone use, as well as withdrawal-like symptoms when unable to use their phone. Finally, individuals who reported more symptoms of problematic mobile phone use on the PUMP Scale were more likely to endorse feeling “addicted” to their cellular phone. These findings support the popular construct of problematic mobile phone use (sometimes referred to as “cell phone addiction”) and suggest that this area merits further study.

When considering these findings, it is important to acknowledge some limitations of the present study. First, like many instrument development studies, the sample was relatively small (*N* = 244) and was comprised of individuals recruited through convenience. Many of the participants were recruited from a college campus, and some were recruited from a mobile phone store, which may have introduced selection bias. Thus, the generalizability of results to the population as a whole may be limited. Future validation efforts should include larger, more diverse samples that are randomly selected. Second, the use of self-report, particularly regarding past behaviors and experiences, may have introduced biases due to faulty recall, social desirability, or shared methods variance. Obtaining more objective measures (i.e., mobile phone records, collateral reports) would strengthen the data. Third, no “gold standard” measure (i.e., accepted formal diagnostic criteria) exists for problematic mobile phone use. Therefore, it was not possible to assess the operating characteristics of the PUMP Scale. Future research is needed to identify the cut-point(s) of the PUMP Scale for the purpose of detecting clinically significant symptoms. 

 It must also be emphasized that the construct of problematic mobile phone use is not yet well-studied or supported in the literature. The merits and implications of considering this construct should be further explored and developed. Clearly, some individuals use their mobile phones more than others, but the reasons for this may be multifaceted. Job requirements, safety issues, and family and social factors all may contribute. However, “problematic mobile phone use” appears to extend beyond frequency of use, to the extent that mobile phone use produces social, occupational, and psychological distress; it may be useful to identify these symptoms as potential targets for prevention and intervention. More research is needed to support the results of the current study. Finally, future studies should elucidate the mechanisms underlying problematic mobile phone use, in order to determine whether it exists as a primary phenomenon or alternatively is a symptom of other underlying pathology (e.g., anxiety disorders, impulse control deficits, personality factors). The long-term goal of research into problematic mobile phone use should be to effectively identify and treat problem users or those at risk for problematic use and ultimately to maximize communication utility of mobile technology while minimizing resulting dysfunction.

## Figures and Tables

**Table 1:** Pearson correlations between PUMP Scale, CPDQ, CUQ, and self-assessment items.

Item	PUMPS total score	*P *
CPDQ^a^ total score	.755	<.001
CUQ^b,c^		
How frequently do you typically use the email function on your phone?	.225	<.001
How frequently do you typically use the internet feature of your phone?	.312	<.001
How frequently do you use the games feature of your cell phone?	.203	.002
How often do you talk on the phone while driving?	.411	<.001
How often do you write text messages or emails while driving?	.612	<.001
Self-assessment questions		
I sometimes think that I might be “addicted” to my cell phone.	.733	<.001
I use my cell phone more often than other people I know.	.609	<.001
Friends or family members have commented to me about my cell phone use.	.626	<.001

^a^CPDQ refers to the cell phone dependency tendency questionnaire.

^b^CUQ refers to the cell phone use questionnaire.

^c^All CUQ item responses ranged from 1= never to 6= constantly.

**Table 2:** PUMP Scale item analysis.

Item	M (SD)	Range	Corrected item total correlation	Alpha if item deleted
When I decrease the amount of time spent using my cell phone I feel less satisfied. *(Tolerance) *	1.61 (.96)	1–5	.68	.933
Tolerance—I need more time using my cell phone to feel satisfied than I used to need. *(Tolerance) *	1.53 (.84)	1–5	.65	.933
When I stop using my cell phone, I get moody and irritable. *(Withdrawal) *	1.43 (.83)	1–5	.72	.933
It would be very difficult, emotionally, to give up my cell phone. *(Withdrawal) *	2.36 (1.36)	1–5	.62	.934
The amount of time I spend using my cell phone keeps me from doing other important work. *(Longer time than intended) *	1.70 (.99)	1–5	.74	.932
I have thought in the past that it is not normal to spend as much time using a cell phone as I do. *(Longer time than intended) *	1.73 (1.06)	1–5	.57	.934
I think I might be spending too much time using my cell phone. *(Great deal of timespent) *	1.67 (1.09)	1–5	.72	.932
People tell me I spend too much time using my cell phone. *(Great deal of time spent) *	1.60 (1.08)	1–5	.74	.931
When I am not using my cell phone, I am thinking about using it or planning the next time I can use it. *(Craving) *	1.86 (1.07)	1–5	.75	.931
I feel anxious if I have not received a call or message in some time. *(Craving) *	2.23 (1.23)	1–5	.67	.932
I have ignored the people I'm with in order to use my cell phone. *(Activities given up or reduced) *	2.09 (1.28)	1–5	.72	.931
I have used my cell phone when I knew I should be doing work/schoolwork. *(Activities given up or reduced) *	2.73 (1.56)	1–5	.68	.933
I have used my cell phone when I knew I should be sleeping. *(Use despite physical or psychological problems) *	2.66 (1.64)	1–5	.64	.934
When I stop using my cell phone because it is interfering with my life, I usually return to it. *(Use despite physical or psychological problems) *	1.85 (1.10)	1–5	.67	.933
I have gotten into trouble at work or school because of my cell phone use. *(Failure to fulfill role obligations) *	2.00 (1.38)	1–5	.56	.935
At times, I find myself using my cell phone instead of spending time with people who are important to me and want to spend time with me. *(Failure to fulfill role obligations) *	1.65 (.96)	1–5	.74	.932
I have used my cell phone when I knew it was dangerous to do so. *(Use in physically hazardous situations) *	2.45 (1.41)	1–5	.55	.935
I have almost caused an accident because of my cell phone use. *(Use in physically hazardous situations) *	2.13 (1.32)	1–5	.51	.936
My cell phone use has caused me problems in a relationship. *(Use despite social or interpersonal problems) *	1.53 (1.02)	1–5	.43	.936
I have continued to use my cell phone even when someone asked me to stop. *(Use despite social or interpersonal problems) *	1.69 (1.15)	1–5	.56	.934

Note: item responses ranged from 1 = strongly disagree to 5 = strongly agree.

**Table 3:** PUMP Scale item frequencies.

Item	Strongly disagree %	Disagree %	Neutral %	Agree %	Strongly agree %
When I decrease the amount of time spent using my cell phone I feel less satisfied.	62.8	23.0	5.9	7.5	0.8
I need more time using my cell phone to feel satisfied than I used to need.	64.0	23.8	7.5	2.9	0.8
When I stop using my cell phone, I get moody and irritable.	73.2	16.7	4.6	5.0	0.4
It would be very difficult, emotionally, to give up my cell phone.	39.7	18.4	13.8	20.9	6.7
The amount of time I spend using my cell phone keeps me from doing other important work.	59.4	20.9	10.9	8.4	0.4
I have thought in the past that it is not normal to spend as much time using a cell phone as I do.	57.7	18.8	11.7	8.8	1.3
I think I might be spending too much time using my cell phone.	65.7	15.5	7.9	8.4	2.5
People tell me I spend too much time using my cell phone.	70.3	12.6	6.7	7.9	2.5
When I am not using my cell phone, I am thinking about using it or planning the next time I can use it.	50.2	26.8	12.1	8.8	2.1
I feel anxious if I have not received a call or message in some time.	39.8	20.7	19.5	16.6	3.3
I have ignored the people I'm with in order to use my cell phone.	47.3	20.7	13.7	12.4	5.8
I have used my cell phone when I knew I should be doing work/schoolwork.	36.9	10.4	12.0	23.7	17.0
I have used my cell phone when I knew I should be sleeping.	43.2	7.5	10.0	19.1	20.3
When I stop using my cell phone because it is interfering with my life, I usually return to it.	56.1	14.2	20.9	6.3	2.1
I have gotten into trouble at work or school because of my cell phone use.	59.1	10.3	8.3	15.7	6.6
At times, I find myself using my cell phone instead of spending time with people who are important to me and want to spend time with me.	59.0	25.5	7.9	6.3	1.3
I have used my cell phone when I knew it was dangerous to do so.	39.4	14.9	12.9	25.3	7.1
I have almost caused an accident because of my cell phone use.	47.9	17.8	14.9	12.4	7.0
My cell phone use has caused me problems in a relationship.	74.0	11.2	5.0	8.3	1.7
I have continued to use my cell phone even when someone asked me to stop.	67.2	11.6	10.0	7.5	3.7
